# Microaerobic degradation of crude oil and long chain alkanes by a new *Rhodococcus* strain from Gulf of Mexico

**DOI:** 10.1007/s11274-023-03703-3

**Published:** 2023-07-29

**Authors:** Katy Juárez, Lizeth Reza, Luz Bretón-Deval, Daniel Morales-Guzmán, María R. Trejo-Hernández, Fernando García-Guevara, Paloma Lara

**Affiliations:** 1 Departamento de Ingeniería Celular y Biocatálisis, Instituto de Biotecnología, Universidad Nacional Autónoma de México, Av. Universidad 2001. Col. Chamilpa., Cuernavaca, Morelos 62210 México; 2 Departamento de Microbiología Molecular, Instituto de Biotecnología, Universidad Nacional Autónoma de México, Av. Universidad 2001. Col. Chamilpa., Cuernavaca, Morelos 62210 México; 3 Consejo Nacional de Ciencia y Tecnología, Avenida Insurgentes Sur 1582, Crédito Constructor, Ciudad de México, México; 4 Centro de Investigación en Biotecnología, Universidad Autónoma del Estado de Morelos, Av. Universidad 1001. Col. Chamilpa., Cuernavaca, Morelos 62209 México; 5 Centro de Ciencias Genómicas, Universidad Nacional Autónoma de México, Avenida Universidad s/n, Cuernavaca, Morelos 62210 México; 6 Science for Life Laboratory, KTH – Royal Institute of Technology, Stockholm, SE-171 21 Sweden

**Keywords:** *Rhodococcus qingshengii* GOMB7, Hydrocarbon degradation, Gulf of Mexico, Pollution, Bacterial community, Microaerobic conditions, Crude oil degradation

## Abstract

Bacterial degradation of crude oil is a promising strategy for reducing the concentration of hydrocarbons in contaminated environments. In the first part of this study, we report the enrichment of two bacterial consortia from deep sediments of the Gulf of Mexico with crude oil as the sole carbon and energy source. We conducted a comparative analysis of the bacterial community in the original sediment, assessing its diversity, and compared it to the enrichment observed after exposure to crude oil in defined cultures. The consortium exhibiting the highest hydrocarbon degradation was predominantly enriched with *Rhodococcus* (75%). Bacterial community analysis revealed the presence of other hydrocarbonoclastic members in both consortia. In the second part, we report the isolation of the strain *Rhodococcus* sp. GOMB7 with crude oil as a unique carbon source under microaerobic conditions and its characterization. This strain demonstrated the ability to degrade long-chain alkanes, including eicosane, tetracosane, and octacosane. We named this new strain *Rhodococcus qingshengii* GOMB7. Genome analysis revealed the presence of several genes related to aromatic compound degradation, such as *benA*, *benB*, *benC*, *catA*, *catB*, and *catC*; and five *alkB* genes related to alkane degradation. Although members of the genus *Rhodococcus* are well known for their great metabolic versatility, including the aerobic degradation of recalcitrant organic compounds such as petroleum hydrocarbons, this is the first report of a novel strain of *Rhodococcus* capable of degrading long-chain alkanes under microaerobic conditions. The potential of *R. qingshengii* GOMB7 for applications in bioreactors or controlled systems with low oxygen levels offers an energy-efficient approach for treating crude oil-contaminated water and sediments.

**Supplementary Information:**

The online version contains supplementary material available at 10.1007/s11274-023-03703-3.

## Introduction

The Gulf of Mexico (GoM) basin is a rich and varied ecosystem that supports a range of economic activities (Yoskowitz et al. 2016). This region is home to numerous companies engaged in oil and gas exploration and production. Currently, there are more than 10,000 platforms and 27,000 abandoned wells in the north and more than 2,000 offshore wells in the south (Pulster et al. 2020). The Catastrophic Deepwater Horizon spill in 2010, where 4.9 barrels of crude oil from the Macondo well were released into the GoM, resulted in the chronic exposure of organisms to toxic compounds for decades damaged this ecosystem (Love et al. 2013; Kolian et al. 2015; Pulster et al. 2020). Contaminated sediments may harbor microorganisms with exceptional metabolic capabilities that could be harnessed to develop biotechnological processes for cleaning up polluted ecosystems. Several bacterial groups have been shown to degrade hydrocarbons through aerobic pathways, using oxygen as an electron acceptor and co-substrate in mono- or di-oxygenase reactions to oxidize the substrate (Brennerova et al. 2022). Alternatively other bacterial groups may degrade hydrocarbons anaerobically, utilizing diverse final electron acceptors such as nitrate, iron, sulfate, manganese, and chlorate, or even coupled to methanogenesis and fermentation (Holmes and Smith 2016; Laczi et al. 2020; Wegener et al. 2022).

The Gulf of Mexico Research Consortium (Consorcio de Investigación del Golfo de México (CIGoM), a multidisciplinary research group, has focused on understanding the potential environmental impacts of oil spills on marine ecosystems (Godoy-Lozano et al. 2018; Hernández-López et al. 2019; Velez et al. 2019; Cerqueda-García et al. 2020; Raggi et al. 2020; Rodríguez-Salazar et al. 2021; Loza et al. 2022). Since its establishment in 2015, this academic group has conducted several sampling cruises. As part of this study, we analyzed the microbial diversity study from a marine sediment sample and demonstrated the hydrocarbonoclastic activity of some of these indigenous microorganisms. Marine sediments provide environments where transient hypoxia can occur, making them potential habitats for aerobic microorganisms adapted to tolerate hypoxic conditions. The Gulf of Mexico has been chronically exposed to hydrocarbons derived from petroleum. Therefore, we hypothesize that it is possible to enrich and isolate bacteria capable of degrading crude oil and pure hydrocarbons under oxygen limitation from a marine sediment sample from the Gulf of Mexico.

The main objective of this study was to explore the hydrocarbon-degrading capabilities of aerobes or facultative anaerobes indigenous microorganisms present in sediments of the Gulf of Mexico (GoM) under microaerobic conditions. In this study we characterized two distinct consortia enriched in microaerobic conditions with crude oil as the only source of carbon and energy. The consortium with the highest hydrocarbon degradation was predominantly enriched with *Rhodococcus* spp, the second consortium was enriched with *Marinobacter* and *Alcanivorax*. However, the hydrocarbon degradation efficiency with the second consortium was minimal under microaerobic conditions. The *Rhodococcus* strain that degrades hydrocarbons under microaerobic conditions was isolated and subsequently sequenced and characterized.

There are several reports pointing out that *Rhodococcus* exhibits great metabolic versatility, including the ability to degrade hydrocarbons under aerobic conditions (Li et al. 2013; Auta et al. 2018; Gao et al. 2020; Peng et al. 2020a, b; Zampolli et al. 2020; Delegan et al. 2022; Thi Mo et al. 2022). However, as far as we know, this is the first report of hydrocarbon degradation under microaerobic conditions. Our culture conditions can be a reference for the improvement of degradation processes in low oxygen environments and of other previously studied *Rhodococcus* strains.

## Materials and methods

### Sediment collection

The sample used in this study was collected in the Northwestern Gulf of Mexico in May 2017 at a depth of 1,374 m from station B7 (25°40′52.3′′N 95°35′62.9′′W) in the Perdido Fold area during “Metagenomic 2” campaign. A sub-core 30 cm was aseptically sampled from a Hessler-Sandia MK-II box core (40 × 40 cm) from which (0–5 cm) subsamples were taken for this study. The sediment sample was sealed with food grade plastic and transported at 4 °C to the laboratory and stored at the same temperature in the dark until processing. The sediment core was not aerated, sifted, or dried.

### Media used for isolation and bacterial enrichment

The culture media MMSw by Marine Medium Shewanella, is a modified culture media used to isolate facultative microorganisms contains NH_4_Cl 1.49 g/L, KCl 0.099 g/L, NaH_2_PO_4_ 0.599 g/L, Na_2_SO_4_ 0.099 g/L, MgSO_4_*7H_2_O 0.246 g/L, CaCl_2_ 0.022 g/L, PIPES 6.04 g/L, Na_2_SeO_4_ 1 mM and NaCl 21 g/L, it was supplemented with 10 ml/L of two solutions DL Minerals and DL vitamins reported by Coppi et al. (2001). The culture media MARS from Marine water Supplemented, designed by us for enrich microorganism able to growth in sea water with selected supplements, it contains water collected from the Perdido fold area, sterilized by filtration and supplemented with NH_4_Cl 0.19 g/L as nitrogen source and K_2_HPO_4_ 0.006 g/L, KH_2_PO_4_ 0.012 g/L as phosphate source, 10 ml/L of DL Minerals solution and DL Vitamins solution (Coppi et al. 2001). Both media were supplemented with acetate 20 mM or hydrocarbon as carbon sources.

The composition of the DL trace mineral solution per liter of deionized water is 1.5 g of nitriloacetic acid, 3.0 g of MgSO_4_, 0.5 g of MnSO_4_*H_2_O, 1 g of NaCl, 0.1 g of FeSO_4_*7H_2_O, 0.1 g of CaCl_2_*2H_2_O, 0.1 g of CoCl_2_*6H_2_O, 0.13 g of ZnCl_2_, 0.01 g of CuSO_4_*5H_2_O, 0.01 g of AlK(SO_4_)_2_*12H_2_O, 0.01 g of H_3_BO_3_, 0.025 g of Na_2_MoO_4_*2H_2_O, 0.024 g of NiCl_2_*6H_2_O and 0.025 g of Na_2_WO_4_*2H_2_O (Coppi et al. 2001).

The composition of the DL vitamin solution per liter of deionized water is 0.0002 g of biotin, 0.005 g of calcium pantothenate, 0.0001 g of vitamin b12, 0.005 g of para-aminobenzoic acid, 0.005 g of thioctic acid, 0.005 g of nicotinic acid, 0.005 g of thiamine hydrochloride, 0.005 g of riboflavin, 0.01 g of pyridoxine hydrochloride and 0.002 g folic acid (Coppi et al. 2001).

### Microbial consortia enrichment

Microaerobic hydrocarbonoclastic bacteria were enriched as follows: 0.5 g of marine sediment from station B7 was added to 200 ml crimp-top Teflon serum bottles containing 50 ml of mineral salt medium (MMSw and MARS). The medium was supplemented with crude oil API 40, at a concentration of 860 ppm (mg/L), cultures were incubated at 30 °C without agitation and every 8 days 10% (v/v) of the enriched cultures were transferred to 50 ml of fresh medium with crude oil, for six consecutive transfers. Finally, the cultures were cryopreserved with 10% Sigma DMSO (Dimethyl Sulfoxide) at -65 °C.

Microbial consortia crude oil degradation (8600 ppm of crude oil API 40), were performed in microaerobic conditions. Cryopreserved aliquots were directly inoculated in 50 ml of MMSw or MARS culture medium in 100 ml crimp-top teflon serum bottles. The bottles were kept static with an air volume of 0.0593 g/mol. The experiments and abiotic controls were carried out in triplicate.

### Strain isolation and culture

To obtain culturable bacterial strains, 0.5 g of marine sediment was added to 50 ml of MMSw culture medium. The mixture was serially diluted and spread onto solid MMSw medium with acetate 20 mM. The plates were then incubated at 30 °C. Plating and incubations were carried out inside an anaerobic chamber with an atmosphere containing 7% H_2_, 10% CO_2_ and 83% N_2_, all at 30 °C. Picked colonies were restreaked onto solid MMSw medium with acetate 20 mM until their isolation.

### Crude oil degradation in two conditions

The degradation of crude oil API 40 (8600 ppm) by *R. qingshengii* GOMB7 was evaluated under two different conditions. Firstly, the strain was grown aerobically using 50 ml of MMSw medium in a 250 Erlenmeyer flask with agitation at 200 rpm and gaseous interchange. Secondly, a microaerobic culture was performed using 50 ml of MMSw medium in 100 ml crimp top teflon serum bottles with 0.0593 g/mol of air and without agitation. Experiments and abiotic controls were conducted in triplicate.

### Hydrocarbon quantification

The cultures were subjected to three extractions with dichloromethane and dried with Na_2_SO_4_. The remaining hydrocarbon content was quantified through gravimetry. Subsequently, the samples were analyzed using a Hewlett Packard HP 5890 Series II Gas Chromatograph equipped with a flame ionization detector (FID) and a Phenomenex ZB-5 rubber capillary column of (30 m long x 0.32 mm inner diameter; 0.25 μm film). Sample injection was performed with 1:10 split ratio under the following conditions: helium as the carrier gas, injection temperature set at 270 °C, detector temperature at 330 °C, and the furnace initially set at 60 °C for 2 min. The temperature was then ramped up at a rate of 6 °C/min to 250 °C (ramp1) and further increased at a rate of, ramp2 from 12 °C /min up to 320 °C, isotherm 10.5 min; execution time 50 min.

### 16S rRNA amplification, sequencing, and analysis

The microbial community composition of a sediment sample from B7 and cryopreserved bacterial enrichments was determined through high throughput 16S rRNA gene sequencing. Genomic DNA was isolated with the DNeasy PowerSoil kit (QIAGEN), and the variable region of the 16S rRNA gene was amplified using the primer pair S-D-Bact-0341-b-S-17/S-D-Bact-0785-a-A-21 (Klindworth et al. 2013). PCR conditions were 98°-3 min, 98°-30 s/60°-30 s/72°-30 s for 25 cycles and 72°-5 min using Phusion Polymerase (Thermo Scientific). Library preparation was performed using the Nextera XT DNA Library Prep Kit (Illumina) following Illumina’s protocol. The libraries were sequenced on an Illumina MiSeq platform. Amplicon sequences were processed using dada DADA2 with default parameters (Callahan et al. 2016), and the resulting Amplicon Sequence Variants (ASVs) were taxonomically annotated using Vsearch against the Silva-132 database. To ensure sufficient sequencing depth, alpha-rarefaction analysis was performed using qiime2 v2022.2 (Bolyen et al. 2019). The raw data have been uploaded into NCBI with the BioSamples accession numbers SAMN29405029, SAMN29405030 and SAMN29405031.

### Statistical analysis

Crude oil and long-chain alkane degradation experiments were conducted using consortia and the isolated strain, with three biological replicates for each assay. Controls were also analyzed in triplicate. The mean and standard deviation were calculated based on the triplicate results. An ANOVA analysis was performed on the degradation assays to determine statistically significant differences between the assays inoculated with *R. qingshengii* GOMB7 and the abiotic controls.

### Genome bioinformatic analysis

Genomic DNA from the strain GOMB7 was extracted using the DNeasy PowerSoil kit (QIAGEN). Paired-end libraries were sequenced using an Illumina GAIIX at the University Massive DNA Sequence Unit, Instituto de Biotecnología, Universidad Nacional Autónoma de México (UUSMD-IBT, UNAM). Shotgun paired end sequences were assembled using five different assemblers independently, MIRA v. 5.1 (Chevreux et al. 2004), MaSuRCA v. 3.4.2 (Zimin et al. 2013), SPAdes v. 3.13.0 (Nurk et al. 2013), IDBA v. 1.1.3 (Peng et al. 2012) and Megahit v. 1.2.9 (Li et al. 2016) with default parameters. The five assemblies were refined into one final assembly using GenomeFinisher v. 1.4 (Guizelini et al. 2016). The final sequence was uploaded into NCBI (BioProject: PRJNA732393). The annotation was performed using the NCBI Prokaryotic Genome Annotation Pipeline (PGAP) (https://www.ncbi.nlm.nih.gov/genome/annotation_prok/).

Ten genomes annotated with *Rhodococcus* were used to conduct a pangenomics analysis using the Anvio workflow (Delmont and Eren 2018). The analysis utilized the extension of PyANI (Pritchard et al. 2016) to compute the average nucleotide identity across the genomes and sour mash (Brown and Irber 2016) to calculate the mash distance (Figure S2). The GenBank accession numbers for the assembled *Rhodococcus* genomes were: GCA_001894885, GCA_004011835, GCA_004011825, GCA_018729485, GCA_008297975, GCA_900478115, GCA_004011865, GCA_000470885, GCA_002863905 and GCA_001620305 (Fig. 6).

Sequences of proteins were mapped to the Kyoto Encyclopedia of Genes and Genomes database (KEGG) using the Automatic Annotation Server (KAAS v2.1) and the single-directional best hit method (SBH). The annotated genes were mapped against the genes involved in the follow pathways: xylene, fluorobenzoate, chlorocyclohexane, chloroalkane, aminobenzoate, toluene, dioxin, ethylbenzene, styrene, atrazine, naphthalene, and benzoate degradation. The pathways related to the hydrocarbon degradation were show in a heatmap using R, aiming to elucidate the strategies employed by different *Rhodococcus* strains in handling hydrocarbon pollution.

## Results and discussion

### Microbial community of deep sediment sample from Northwest of GoM

Marine sediments from areas chronically exposed to crude oil are a plentiful source of microorganisms with exceptional metabolic capabilities for degrading recalcitrant compounds. These microorganisms can be utilized for waste treatment in the oil industry, thereby mitigating its environmental impact. In this study, microbial enrichment and strain isolation were performed using marine sediments collected at a depth of 1,374 m from station B7 (25°40′52.3′′N 95°35′62.9′′W) in the GoM. A portion of this sample was subjected to DNA extraction, followed by 16S rRNA amplicon sequencing to analyze bacterial diversity.

**Fig. 1:**
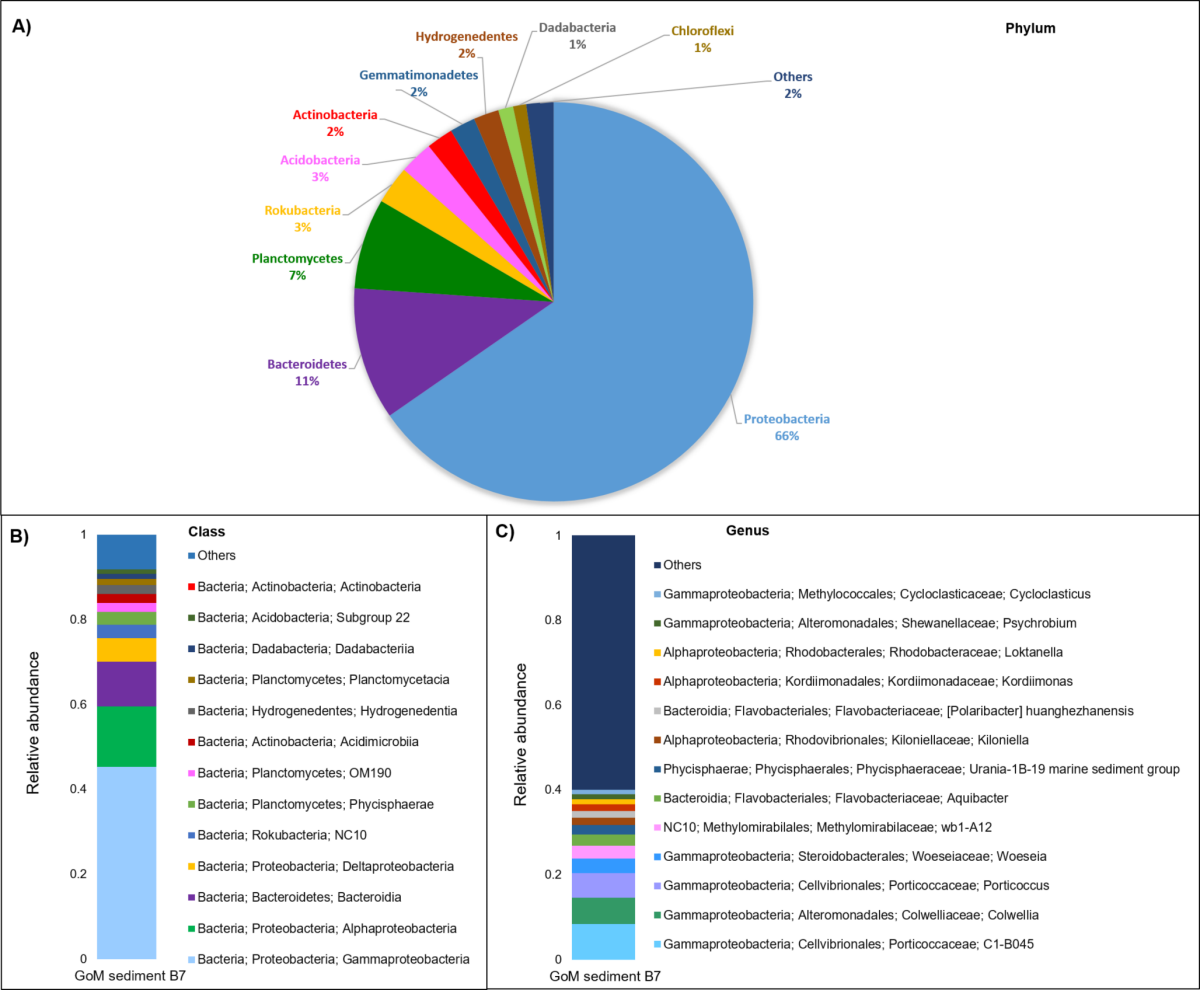
Bacterial community composition in the GoM sediment sample from B7 station. Plots display relative abundances for the 16S rRNA amplicon taxonomic annotations from the B7 sediment sample at **A**) Phylum, **B**) Class, and **C**) Genus levels. Groups with low relative abundances collapsed into the “others” category

The taxonomic assignment of 16S rRNA amplicons is depicted in Fig. 1. Sample B7 exhibited a predominance of Proteobacteria (66%), Bacteroidetes (11%), Planctomycetes (7%), Rokubacteria (3%), Acidobacteria (3%), Actinobacteria (2%), Gemmatimonadetes (2%) and Hydrogenedetes (2%) at the phylum level (Fig. 1A). At the class level, Proteobacteria was represented by Gammaproteobacteria (45%), Alphaproteobacteria (14%), Deltaproteobacteria (5%), while Bacteroidetes were represented by Bacteroidia (10%) and Planctomycetes by Phycisphaerae (3%), OM190 (2%) and Planctomycetacea (1%) (Fig. 1B). The most abundant genus in this sediment sample was C1-B045, belonging to the Class Gammaproteobacteria, representing 8% of the bacterial community. Although poorly characterized, this genus has been proposed to have potential for PAH biodegradation, as shown in reports of microcosm assays with crude oil and seawater, in which C1-B045 is increased 167.4-fold during PAH oxidation (Peng et al. 2020a, b). Other genera such *Colwellia* (6%), *Porticoccus* (6%), and *Kordimonas* (2%), known as hydrocarbon degraders, accounted for 14% of the B7 sediment, and their presence has been reported in previous studies in the same area (Sánchez-Soto Jiménez et al. 2018; Raggi et al. 2020; Ramírez et al. 2020). The genus *Cycloclasticus* was found with a 1% of relative abundance, microorganisms of this genus have been reported to degrade polyaromatic hydrocarbons and are named “obligate hydrocarbonoclastic bacteria’’ (Kasai et al. 2002; Yakimov et al. 2007; Wang et al. 2018).

### Crude oil-degrading bacterial community enrichment

We utilized two different media, MMSw and MARS, to enrich hydrocarbonoclastic bacteria from the B7 deep sediment sample under microaerobic conditions, with crude oil API 40 serving as the sole carbon source. After six subculture steps, the enriched bacterial community was analyzed and cryopreserved for future studies.

**Fig. 2:**
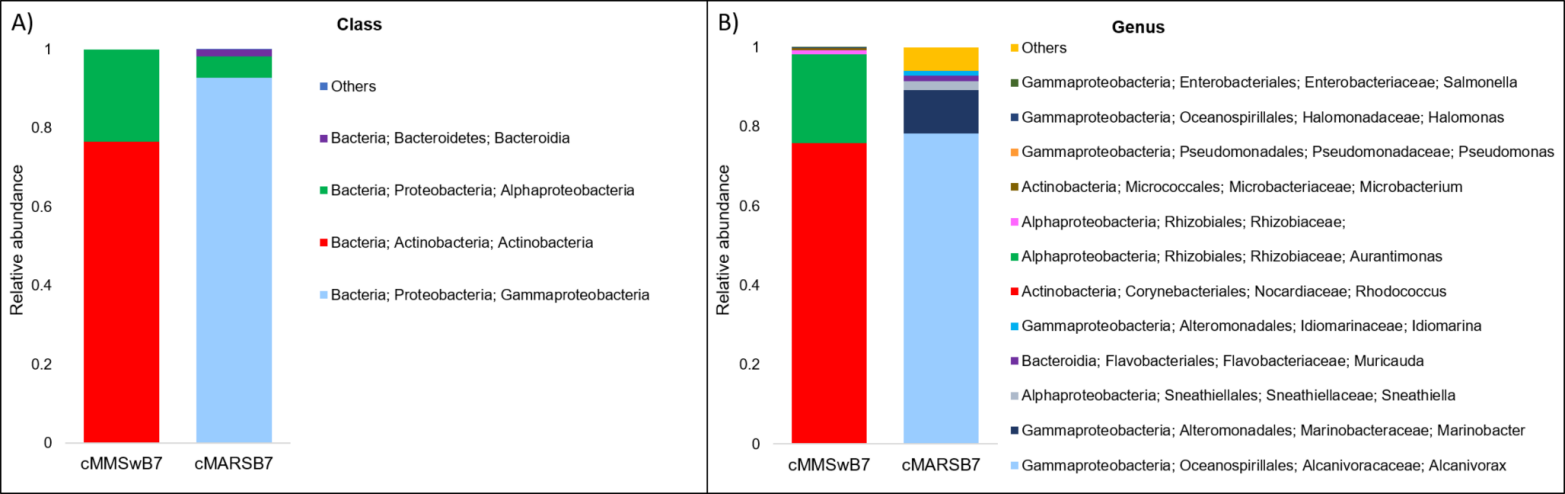
The composition of bacterial consortia. Bar plots displaying relative abundances for the 16S rRNA taxonomic annotation from consortia grown in MMSw and MARNP media. **A**) Class level and **B**) Genus’s level. Groups with low relative abundances collapsed into the “others’’ category

The taxonomic assignment with 16S rRNA amplicons of both consortia is displayed in Fig. 2. Microbial diversity was evaluated using Shannon indexes, which yielded a value of 5.9 for the original sediment, and 4 and 3.4 for cMMSwB7 and cMARSB7, respectively.

In the cMMSwB7 consortium, the classes Actinobacteria (76.3%), Alphaproteobacteria (23.5%) and Gammaproteobacteria (0.2%) were identified (Fig. 2A). At the genus level, *Rhodococcus* (75.9%) was the most abundant, followed by *Aurantimonas* (22.3%), *Microbacterium* (0.4%), *Pseudomonas* (0.1%), *Hallomonas* (0.1%) and *Salmonella* (0.1%) (Fig. 2B). *Rhodococcus* is known for its hydrocarbonoclastic capabilities under aerobic conditions, but not microaerobic conditions (Li et al. 2013; Auta et al. 2018; Gao et al. 2020; Peng et al. 2020a, b; Zampolli et al. 2020; Delegan et al. 2022; Thi Mo et al. 2022). In the cMMSwB7 consortia, *Aurantimonas* was also present, this genus is reported to be involved in Mn-cycling and has been found in bacterial communities associated with hydrocarbon-contaminated regions. It also has been isolated from oxic/anoxic boundaries of marine environments (Anderson et al. 2009).

In the cMARSB7 enriched consortium, the class Gammaproteobacteria (92.8%) was dominant, while Alphaproteobacteria (5.4%) and Bacteroidia (1.7%) were enriched in minor proportions (Fig. 2A). At the genus level, *Alcanivorax* (78%) was the most abundant, followed by *Marinobacter* (10%), *Sneathiella* (2%), *Idiomarina* (1%), and *Muricauda* (1%) (Fig. 2B). There are several reports where strains from the genera *Alcanivorax*, *Marinobacter*, and *Idiomarina* have been identified with the capability to degrade different hydrocarbon compounds aerobically (Wang et al. 2011; Chernikova et al. 2020).

**Fig. 3:**
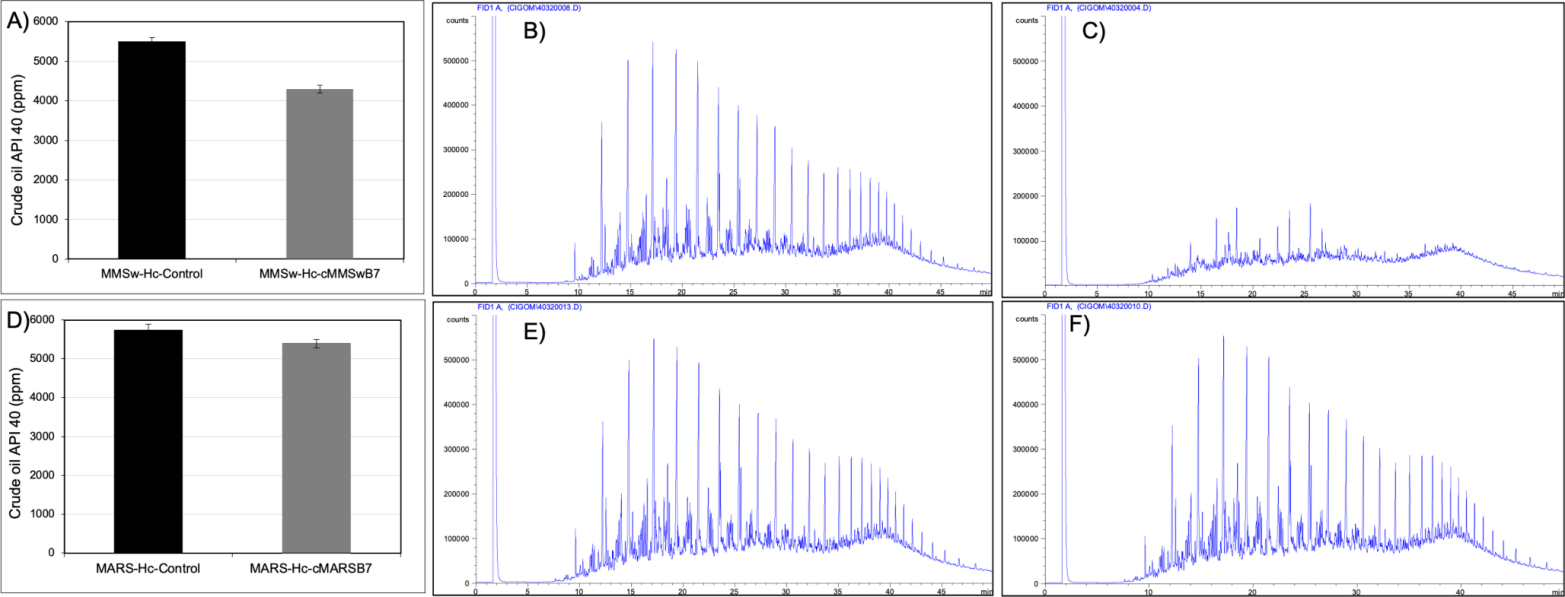
Crude oil degradation by the consortia cMMSwB7 and cMARNPB7. **A**) oil hydrocarbon degradation by cMMSwB7 consortium determined by gravimetric. **B**) hydrocarbon profile determined by GC-FID in abiotic controls in medium cMMSw, **C**) hydrocarbon profile determined by GC-FID in cultures of MMSwB7 consortium. **D**) oil hydrocarbon degradation by cMARNPB7 consortium determined by gravimetric. **E**) hydrocarbon profile GC-FID in abiotic controls in medium MARNP, **F**) hydrocarbon profile determined by GC-FID in cultures of cMARNPB7 consortium. Each GC-FID profile corresponds to one of the biological triplicates, which displayed the same profile

Degradation of crude oil (1% v/v API 40) was evaluated by both consortia studied. After a 28-day incubation period at 30 °C under microaerobic conditions, the cMMSwB7 consortium exhibited a remarkable degradation of 22% (Fig. 3A). In contrast, the cMARSB7 consortium only achieved a degradation of 6%, as determined by gravimetric quantification. These findings highlight the significantly superior performance of the cMMSwB7 consortium in the degradation of API 40 crude oil compared to the cMARSB7 consortium. GC-FID analysis revealed a reduction of various peaks corresponding to different hydrocarbons in cMMSwB7 consortium extracts compared with control samples (Fig. 3B-C). In contrast, no differences were observed in cMARSB7 extracts, as shown in Fig. 3E-F. Despite containing hydrocarbonoclastic bacteria, the degradation of crude oil by cMARSB7 consortium was poor under the microaerobic conditions tested in this study (Fig. 3D-F).

Révész et al. (2020) conducted a study on the effect of oxygen on the bacterial community structure using a biofilm sample collected from groundwater well contaminated with gasoline. They found that the relative abundance of the genus *Rhodococcus* with 5.5% in the aerobic enrichment, while in the microaerobic enrichment, was less than 0.5%. This suggests that oxygen limitation had a negative effect on the *Rhodococcus* metabolism. In a different report, *Rhodococcus* was enriched in a microcosm experiment under hypoxic/anoxic conditions with contaminated sediments during naphthalene degradation (Wilhelm et al. 2018). These contrasting reports suggest important differences in the effect of oxygen limitation on the growth and hydrocarbon degradation capabilities of *Rhodococcus* species.

### *Rhodococcus qingshengii* GOMB7 isolation and characterization

To isolate hydrocarbonoclastic bacteria from sediment B7, serial dilutions of sediment suspension were plated on solid MMSw culture medium supplemented with acetate 20 mM. Isolated colonies were subcultured in MMSw supplemented with 1% v/v (8.6 g/L) crude oil API 40. The isolated strain, *Rhodococcus* sp. strain GOMB7, was the only one capable of growing under these conditions and was further characterized. The hydrocarbonoclastic activity of *R. qingshengii* GOMB7 was tested under microaerobic conditions to compare it with the consortia. Since microorganisms belonging to the *Rhodococcus* genus are reported to be aerobic, the degradation of crude oil by GOMB7 strain was also assayed under aerobic conditions. Fresh cultures of *R. qingshengii* GOMB7 grown in MMSw-acetate were used to inoculate 500 ml of MMSw culture medium supplemented with 1% v/v (8.6 g/L) crude oil API 40. Hydrocarbon degradation was quantified after 28 days of incubation at 30 °C under aerobic and microaerobic conditions.

**Fig. 4:**
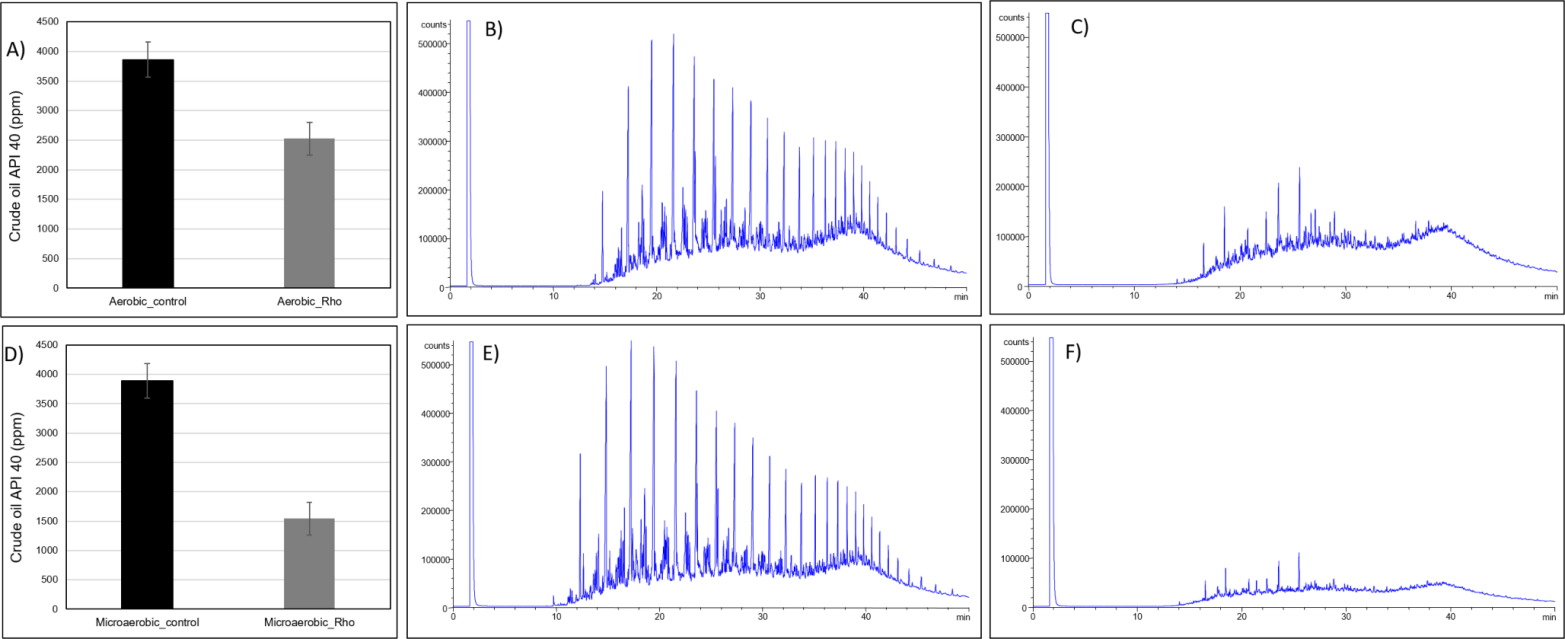
Degradation of crude oil API 40 by *Rhodococcus qingshengii* GOMB7 **A**) aerobic oil hydrocarbon degradation by cMMSwB7 consortium determined by gravimetric. **B**) hydrocarbon profile determined by GC-FID in abiotic controls in aerobic cMMSw medium, **C**) hydrocarbon profile determined by GC-FID in aerobic cultures of *R. qingshengii* GOMB7 strain. **D**) microaerobic oil hydrocarbon degradation by cMMSwB7 consortium determined by gravimetric. **E**) hydrocarbon profile determined by GC-FID in abiotic controls in microaerobic cMMSw medium, **F**) hydrocarbon profile determined by GC-FID in microaerobic cultures of *R. qingshengii* GOMB7 strain. Each GC-FID profile corresponds to one of the biological triplicates, which displayed the same profile

**Fig. 5:**
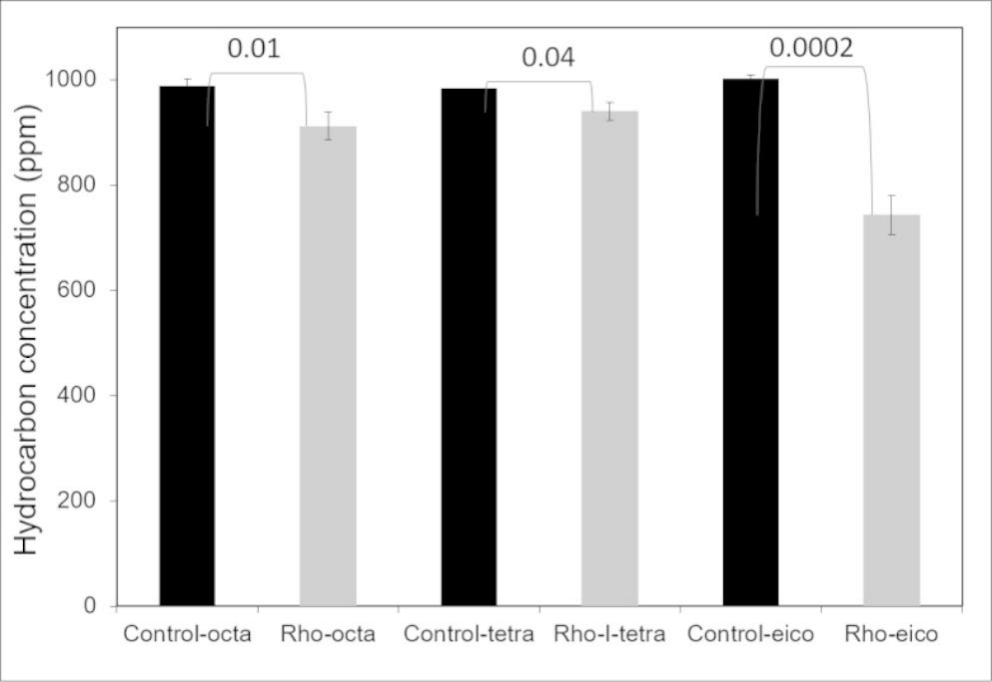
Degradation of the paraffin eicosane, tetracosane, and octacosane by *Rhodococcus qingshengii* GOMB7. Data from gravimetric determinations. P-values from one-way ANOVA analysis are indicated for each pair of assay-abiotic control

Gravimetric quantification showed that *R. qingshengii* GOMB7 degraded 34.6% and 60.4% of total hydrocarbons from crude oil under aerobic and microaerobic conditions, respectively (Fig. 4A and D). The highest degradation was observed under microaerobic conditions compared to aerobic, as demonstrated by GC-FID analysis (Fig. 4B-C, 4E and 4F). In the chromatograms from the GC-FID analysis (Fig. 4), it is evident that *R. qingshengii* GOMB7 can degrade different hydrocarbons, particularly the aliphatic ones, which are more pronounced at the higher weight region (on the right side). To quantify the ability of *R. qingshengii* GOMB7 to degrade long chain alkanes (paraffins), the strain was inoculated in MMSw supplemented with eicosane, tetracosane or octacosane 1 g/L. Hydrocarbon degradation was quantified after 48 h of incubation at 30 °C under microaerobic conditions. *R. qingshengii* GOMB7 was able to degrade 7%, 4% and 25% eicosane, tetracosane or octacosane, respectively, demonstrating its activity on pure paraffins (Fig. 5).

In addition, *R. qingshengii* GOMB7 is able to use glycerol, citrate, acetate, casamino acids, and to a lesser extent lactate and sucrose as carbon sources for growth in MMSw medium (Figure S1).

### Genome analysis *Rhodococcus qingshengii* GOMB7 and identification of genes involved in hydrocarbon degradation

The genome of *R. qingshengii* GOMB7 was sequenced using the Illumina GAIIX platform. The genome assembly resulted in 38 contigs, with an expected genome size of 6,639,181 bp. The N50 value was 491,927, and the L50 value was 5. The sequence genome was deposited in the GenBank with the accession number JAHHZG000000000.1. Genome-wide analysis indicates that *R. qingshengii* GOMB7 belongs to the *R. qingshengii* species, with an average nucleotide identity (ANI) of 99%.

The genome of *R. qingshengii* GOMB7 contains the *catABC-benABCDE* clusters, which is potentially involved and degradation of benzoate (Figure S3), the key intermediate in the degradation of aromatic compounds degradation (Zampolli et al. 2020). This cluster consists of three genes encoding putative transcriptional regulators. The polypeptides encoded by *benA, benB, benC, benD* and *benE* genes share 81%, 85%, 76%, 79% and 75% of identity, respectively, with their homologs in *R. opacus* R7 (Table S1), which were upregulated during xylene degradation (Zampolli et al. 2020). Additionally, we identified five genes in the genome of *R. qingshengii* GOMB7 that encode homologs of alkane 1-monooxygenase AlkB (AIA09965.1) from *R. opacus* B-7. These could be involved in the capability of this strain to degrade various long chain alkanes.

**Fig. 6:**
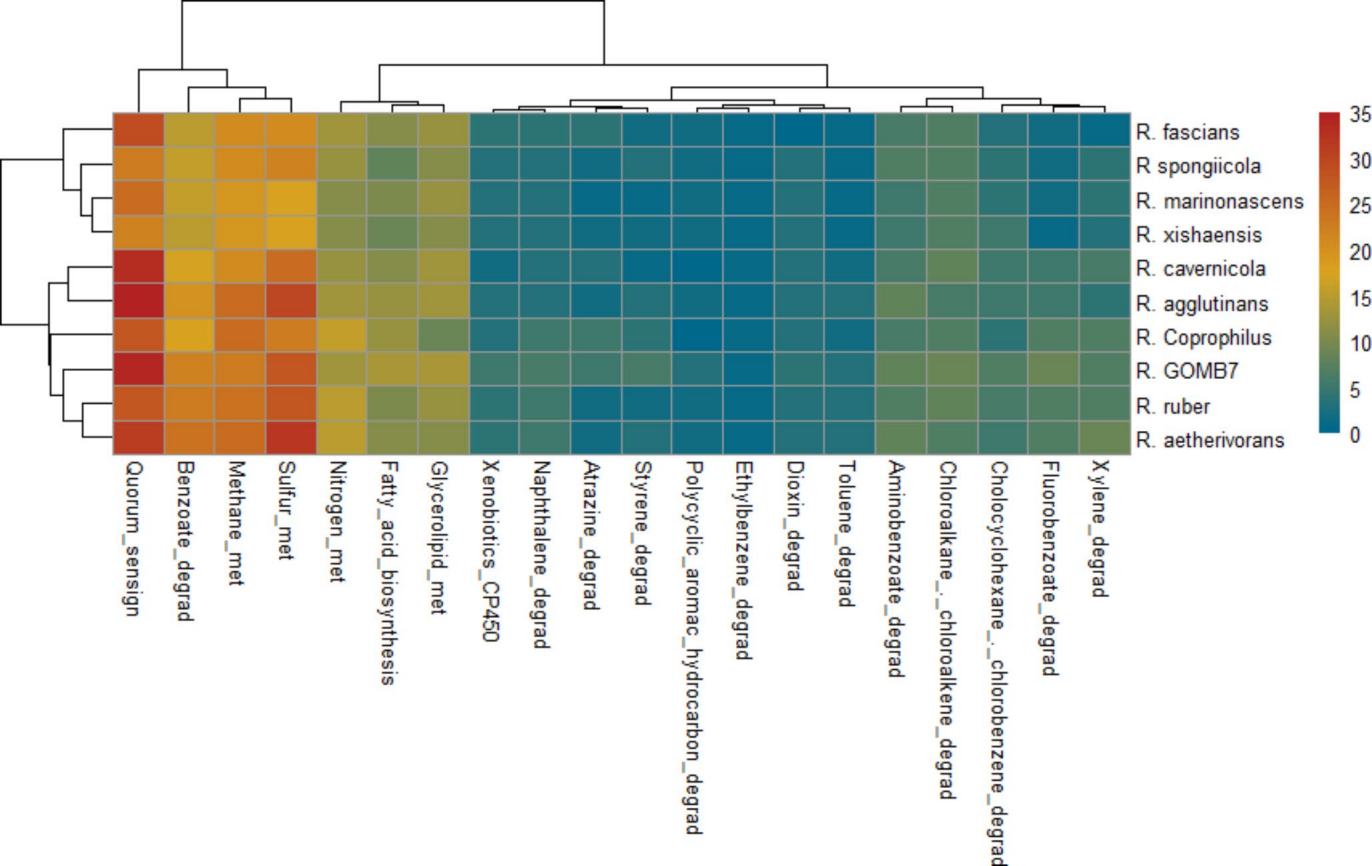
The number of genes in the metabolic pathways related to crude oil degradation present in the closest strains associated with *Rhodococcus qingshengii* GOMB7

To elucidate the different global strategies employed by *Rhodococcus* for degrading crude oil, we conducted a comparative analysis using published assemblies as well as our own assembly. The result showed that not all species of *Rhodococcus* have the same metabolic potential (Fig. 6). The bioinformatic analysis showed differences in the number of genes involved in several pathways that could be directly or indirectly related with the degradation of aromatic compounds (Fig. 6). *R. fascians, R. sponglicola, R. marinonascens*, and *R. xishaensis* exhibit a lower number of genes involved in xylene and fluorobenzoate removal. Conversely, *R. aetherivorans* appears to be a more proficient xylene degrader, while *R*. *qingshengii* GOMB7 could be the better fluorobenzoate degrader, following the logic that it has more genes related to the degradation of the compound.

A successful microorganism in bioremediation, such as *R. qingshengii* GOMB7, possesses various genes that enable the handling of pollutants through alternative pathways, as indicated by bioinformatic analysis. *R. qingshengii* GOMB7 harbors genes involved in the metabolism of styrene, atrazine benzoate, and naphthalene. Additionally, it possesses genes for the non-specific removal of pollutants, such as sulfur and methane pathways. *R. aetherivorans* has a greater number of genes related to those pathways. However, *R. qingshengii* GOMB7 has a higher number of genes involved in fatty acid biosynthesis and glycerolipid metabolism, which could lead to the production of larger quantities of high molecular weight bioemulsifiers that solubilize the compounds and make them more bioavailable. Another exciting result depicted in Fig. 6 is the abundance of genes involved in quorum sensing (QS) of *R. qingshengii* GOMB7. It is well known that QS coordinates the communication among microorganisms, facilitating their response to specific situations or environments by regulating several genes (Urvoy et al. 2022). These genes govern processes such as sporulation, biofilm formation, siderophore formation, bio-surfactant production, and secondary metabolites production, exopolysaccharides production, etc. Through these adaptive mechanisms, microorganisms effectively handle stress conditions and reduce their acclimatization time (Jiménez et al. 2016). Consequently, *R. qingshengii* GOMB7 gains an advantage in utilizing alkanes present in the environment.

In summary, two microbial consortia were enriched using different media and compared for their ability to degrade crude oil under microaerobic conditions. The consortium cultivated in MMSw medium (cMMSwB7) demonstrated the highest crude oil degradation, being *Rhodococcus* the dominant genus. We isolated, sequence and characterized a strain of *Rhodococcus qingshengii* (GOMB7) with the capability to degrade crude oil and paraffins under microaerobic conditions. Genomic analysis confirmed the presence of genes associated with hydrocarbon degradation.

## Conclusion

The marine sediment sample studied from Northwest region of the Gulf of Mexico (GoM) harbors a diverse microbial community, including microorganisms capable of functioning under hypoxic conditions and having the metabolic potential to degrade crude oil as a sole carbon source. These findings suggest that the microbial communities in GoM sediments have adapted to chronic exposure to crude oil and its derivatives, and it is possible to enrich bacterial consortia with biotechnological applications. Notably, the isolated and characterized strain *Rhodococcus qingshengii* GOMB7 exhibited promising degradation capabilities for crude oil and long-chain alkanes in microaerobic conditions. Furthermore, both the isolated strain and the enriched consortia show a great potential for application in the bioremediation of crude oil-polluted wastewater, as well as potential use in bioremediation processes with anoxic sediments.

## Electronic supplementary material

Below is the link to the electronic supplementary material.

Supplementary Material 1: **Fig. S1** Growth of *Rhodococcus qingshengii* GOMB7 with various carbon sources

Supplementary Material 2: **Fig. S2** Pangenomics analysis with the closest strains associated with *Rhodococcus qingshengii* GOMB7

Supplementary Material 3: **Fig. S3***Rhodococcus qingshengii* GOMB7 gene cluster putatively involved in aromatic hydrocarbons degradation. *catA*, encodes a catechol 1,2-dioxygenase; *catB*, encodes a cis,cis-muconate cycloisomerase; *catC*, encodes a muconolactone isomerase; *benA*, encodes a benzoate 1,2-dioxygenase large subunit; *benB*, encodes a benzoate 1,2-dioxygenase small subunit; *benC* encodes an electron transfer component of benzoate 1,2-dioxygenase system; *benD* encodes a 1,6-dihydroxycyclohexa-2,4-diene-1-carboxylate dehydrogenase, *benE*, encodes a benzoate/H(+) symporter; *pvcC*, encodes a pyoverdine chromophore biosynthetic protein

Supplementary Material 4: **Table S1**. Identity percentage between the sequences of proteins involved in bencene degradation in *R. opacus* strain R7 and its homologous in *R. qingshengii* strain GOMB7

## Data Availability

The 16S rRNA sequencing raw data are available at NCBI BioSample under the identifiers SAMN29405029, SAMN29405030 and SAMN29405031. The assembled genome of *Rhodococcus* sp. B7 is available at NCBI BioProject under accession no. PRJNA732393.
