# Supplementary figures and images for: Microaerobic degradation of crude oil and long chain alkanes by a new Rhodococcus strain from Gulf of Mexico
Source: World J Microbiol Biotechnol. 2023 Jul 29;39(10):264. doi: 10.1007/s11274-023-03703-3 (PMC10386958; doi:10.1007/s11274-023-03703-3)

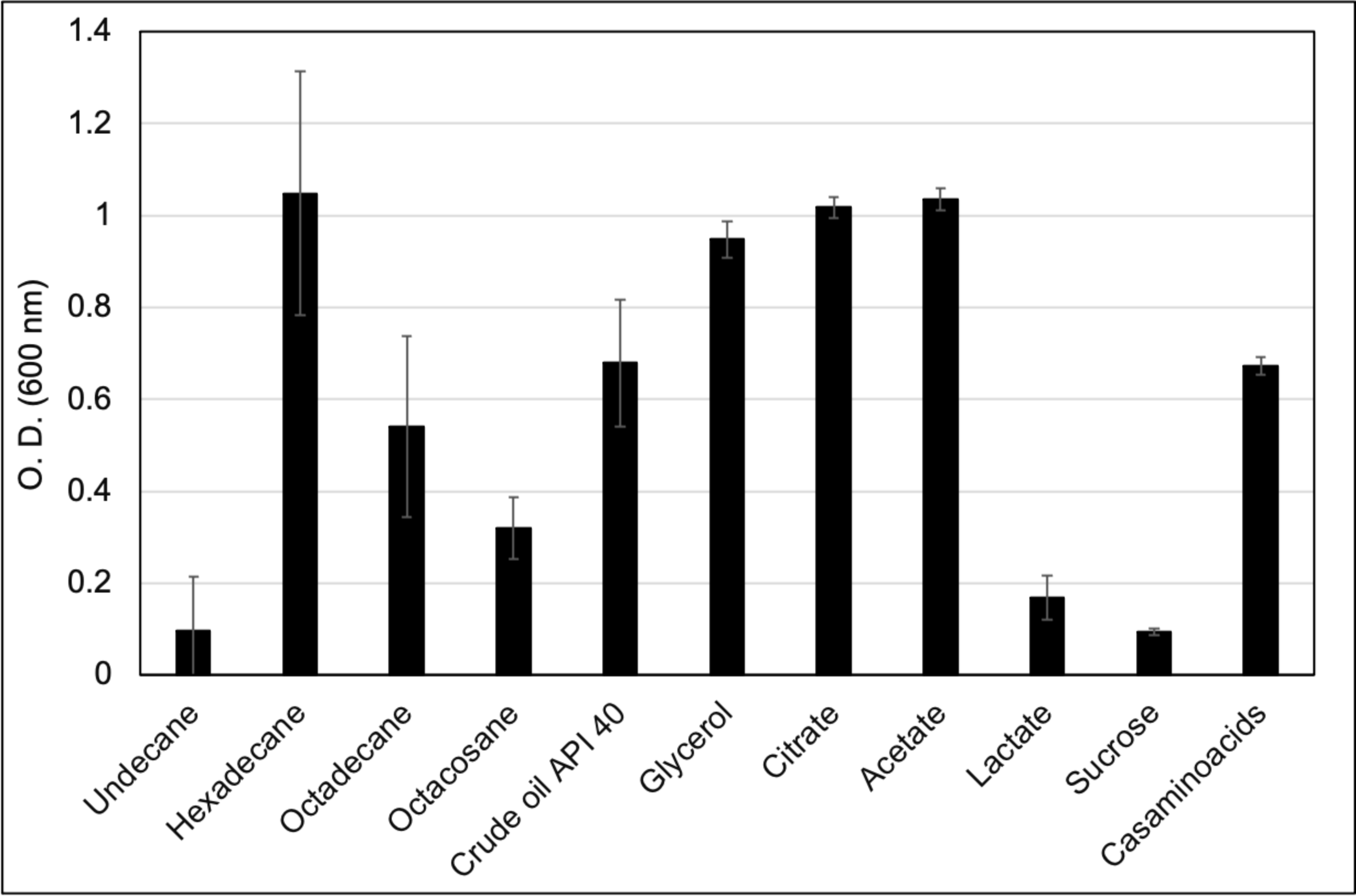

Supplement: Supplementary file 1 — Supplementary Material 1: Fig. S1 Growth of Rhodococcus qingshengii GOMB7 with various carbon sources [file 11274_2023_3703_MOESM1_ESM.png]

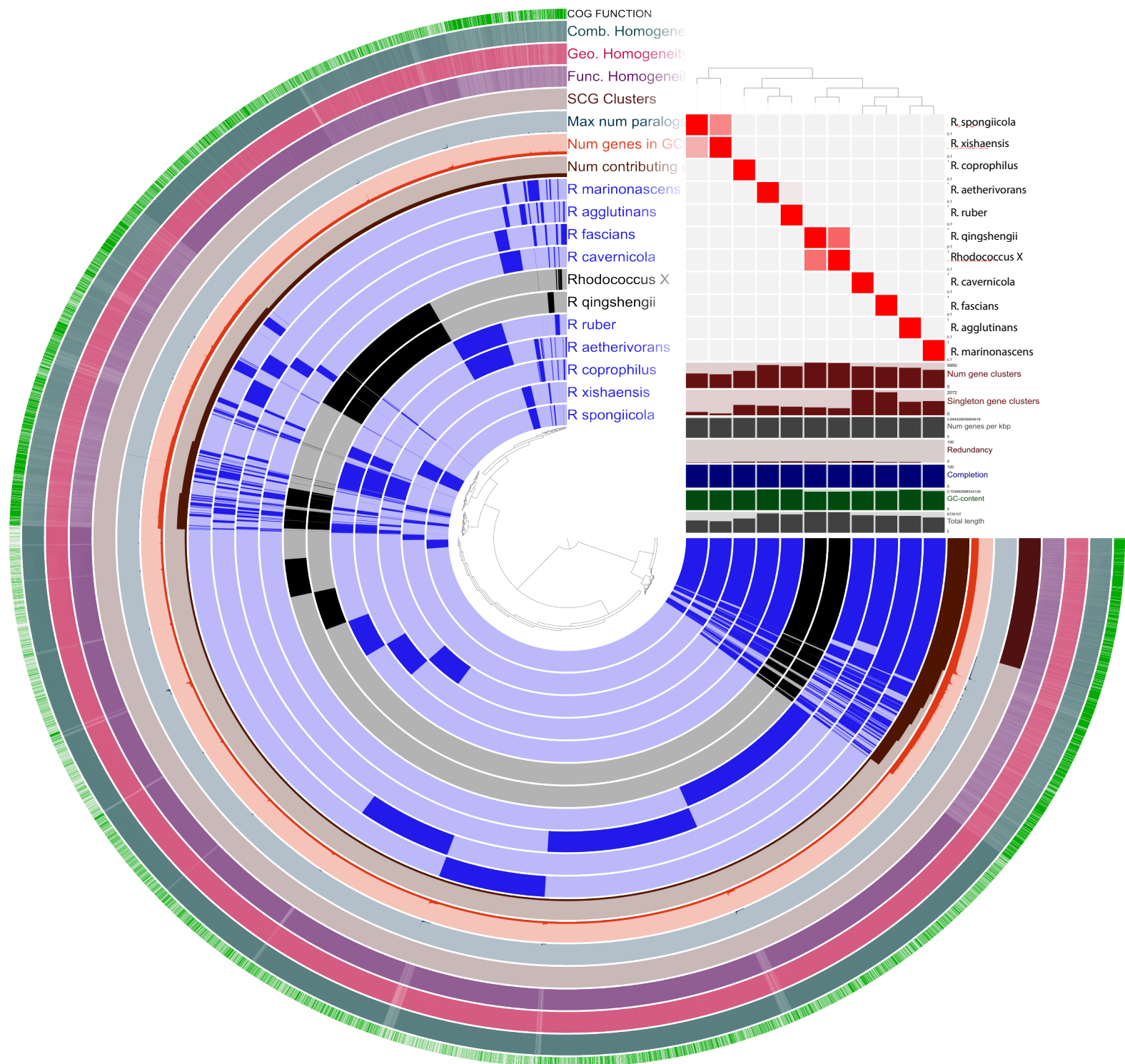

Supplement: Supplementary file 2 — Supplementary Material 2: Fig. S2 Pangenomics analysis with the closest strains associated with Rhodococcus qingshengii GOMB7 [file 11274_2023_3703_MOESM2_ESM.pdf]

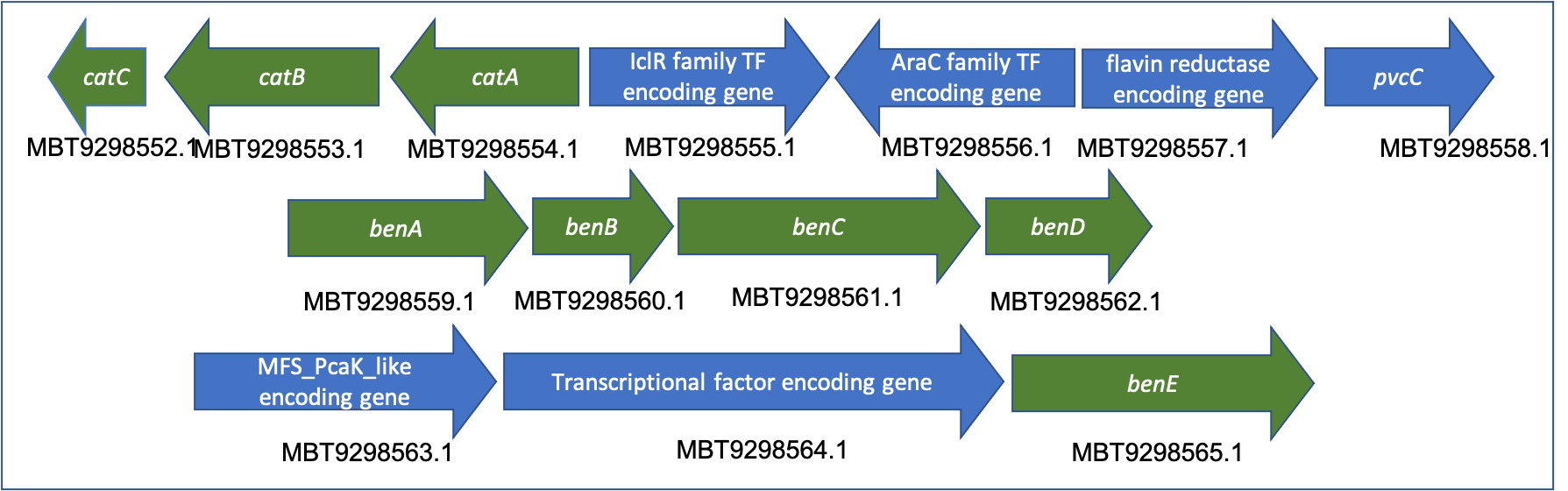

Supplement: Supplementary file 3 — Supplementary Material 3: Fig. S3Rhodococcus qingshengii GOMB7 gene cluster putatively involved in aromatic hydrocarbons degradation. catA, encodes a catechol 1,2-dioxygenase; catB, encodes a cis,cis-muconate cycloisomerase; catC, encodes a muconolactone isomerase; benA, encodes a benzoate 1,2-dioxygenase large subunit; benB, encodes a benzoate 1,2-dioxygenase small subunit; benC encodes an electron transfer component of benzoate 1,2-dioxygenase system; benD encodes a 1,6-dihydroxycyclohexa-2,4-diene-1-carboxylate dehydrogenase, benE, encodes a benzoate/H(+) symporter; pvcC, encodes a pyoverdine chromophore biosynthetic protein [file 11274_2023_3703_MOESM3_ESM.png]
